# Change in Soccer Substitutions Rule Due to COVID-19: Why Only Five Substitutions?

**DOI:** 10.3389/fspor.2020.588369

**Published:** 2021-01-15

**Authors:** Gustavo R. Mota, Izabela Aparecida Santos, Moacir Marocolo

**Affiliations:** ^1^Exercise Science, Health and Human Performance Research Group, Department of Sport Sciences, Institute of Health Sciences, Federal University of Triangulo Mineiro, Uberaba, Brazil; ^2^Exercise Physiology in Health and Human Performance Research Group, Department of Physical Education, University of Uberaba (UNIUBE), Uberaba, Brazil; ^3^Physiology and Human Performance Research Group, Department of Physiology, Federal University of Juiz de Fora, Juiz de Fora, Brazil

**Keywords:** fatigue, coronavirus, football, rules, sports medicine, prophylaxis

## Introduction

Soccer has a high rate and percentage of injuries (Elias, 2001; Wong and Hong, 2005), and more injuries have been observed in soccer than in several other sports (Yde and Nielsen, 1990; Wong and Hong, 2005). Interestingly, the injury rate is markedly higher (~10 times) during matches than training sessions (López-Valenciano et al., 2020), due to several factors such as higher overall demands (e.g., number of contacts/collisions), higher fatigue (e.g., competing against opponents in matches instead of teammates in training) would potentiate these discrepancies (Ekstrand et al., 2011a,b; López-Valenciano et al., 2020). Additionally, the incidence of injuries increases toward the end of each half, indicating that fatigue is involved in injury etiology (López-Valenciano et al., 2020). In training sessions, coaches control the load (e.g., the number of sprints, duration of the session), but load management is much more difficult due to external factors and the nature of the match. Therefore, beyond all previous preparation of the players (e.g., training plan, nutrition, recovery strategies), it is reasonable to consider that “a better” match management (e.g., changes in the rules) might support injury prevention.

From a scientific mindset, the rules of each sport are the cause (independent variable), and the “way to play” is the effect (dependent variable). For example, the recent change in the goal kick rule in soccer: i.e., now the ball no longer has to leave the penalty area before it can be played (FIFA.com, 2020a), has changed the strategies of the teams, and consequently preparation for matches. Analyzing the rules may be fundamental to making rule changes, aiming to enhance a sport (Vamplew, 2007), making the sport safer (e.g., shin guards), healthier (e.g., timeouts for hydration), fairer (e.g., video assistant referee), or more entertaining (i.e., advantageous to sponsors and fans).

Recently we showed that elite soccer has singularly higher overall rule-induced physical demands than other elite team sports, and changes in the substitutions rules might mitigate overall soccer demands (Mota et al., 2020). In March of 2020, soccer seasons were interrupted worldwide to avoid spreading the new coronavirus disease (COVID-19). Leagues resumed the seasons, without fans at the arenas, after several weeks of interruption, causing overlay of schedule. This overlay has caused “even more” congested schedules (e.g., five matches during 14 days), eventually developing accumulated fatigue on players (Silva et al., 2018; Gimenes et al., 2019), and possibly raising injury risk (Ekstrand et al., 2004; Bengtsson et al., 2013; Dellal et al., 2015). To minimize the overload of matches and potential physical issues, the *Fédération Internationale de Football Association* (FIFA) has (temporarily) authorized the increment of up to five substitutions for each team per match instead of three, as per the regular rule (FIFA.com, 2020b). Remarkably, there is limited scientific debate about changes in substitutions rules in soccer which might minimize the impact of congested schedules (exacerbated by the pandemic) and improve injury prevention. Therefore, here we present possibilities to enhance soccer overall, and to improve player welfare, by discussing the substitution rules due to COVID-19 and encouraging constructive discourse.

## Soccer Demands, Fatigue, and Injury Risk

Although sports injuries are multifactorial, accumulated fatigue has an important role (Bengtsson et al., 2013; Gabbett, 2016; Silva et al., 2018). A single soccer match causes fatigue, which is connected with significant match-induced inflammatory responses and muscle damage lasting at least 72 h post-match (Silva et al., 2018). Indeed, Souglis et al. (2015) found greater inflammatory and muscle damage markers after soccer matches than other team sports, concluding that soccer is “the most demanding.” The laws of the game itself, especially the small number of substitutions allowed, is a major contributing factor to the high demands of soccer (Mota et al., 2020). Additionally, soccer has higher total physical demands measured by time-motion studies (e.g., running distances in high-speed), when compared to other team sports (Taylor et al., 2017), and an increase in soccer demands have been revealed over time (Barnes et al., 2014). Research following seven soccer seasons found an impressive increment of ~30% in the sprint distance, as well as technical variables (Barnes et al., 2014). Because high-speed running and injuries are associated (Gabbett, 2016; Buchheit et al., 2019), such physical increment is a concern. Eventually, soccer overload during the player career is linked with a significant loss of time from involvement, early retirement (Knapp et al., 1998), premature osteoarthritis, and a reduced quality of life following retirement (Roos, 1998; Arliani et al., 2014).

Modern soccer has several matches with 72–96 h between matches, generating congested schedules; an issue for clubs and medical staff (Ekstrand et al., 2004; Dellal et al., 2015). Now, with seasons resuming, this issue is exacerbated as most soccer clubs are involved in upwards of two matches each week. We estimate that Liverpool FC (the current World Championship) will face ~25% more matches in comparison to the same period of the prior year (no COVID-19), meaning 25% more load on players, almost certainly increasing injury risk (Gabbett, 2016). For instance, higher muscle injury rates were found in matches with short periods of recovery (≤4 days) vs. longer recovery periods (≥6 days) (Bengtsson et al., 2013). A study showed that two matches/week resulted in a substantially higher injury rate compared with the non-congested period (one match/week), only during the matches (mean 43.3 vs. 18.6). Unlike, when counting both matches and training sessions no differences were found (Dellal et al., 2015), evidence that the “problem” is in the match. Thus, during the “COVID-19 calendar,” most soccer clubs will face a potentially negative injury prevention scenario. In other words, players will face a “high dose” of risk (matches) associated with “low doses” of prevention (i.e., proper fitness training) (Gabbett, 2016; López-Valenciano et al., 2020). Indeed recommendations to return to soccer training and competition after lockdown caused by COVID-19 point out the issues are manifold, such as the loss of performance and the increase of injury risk (Bisciotti et al., 2020). Therefore, we are watching an experiment without a control group!

The potential decrement in injury risk during soccer matches would also benefit team performance. A study following 11 years in the UEFA Champions League found that injuries deteriorate team performance (Hägglund et al., 2013). If the purpose to increase two more substitutions per match in soccer, due to the COVID-19 pandemic, was/is to avoid injuries (FIFA.com, 2020b), why not to do that regularly?

## Which Solutions Do We Have for Such Issues?

Although several strategies (i.e., training and recovery) have been studied and applied to address the issues mentioned (Nédélec et al., 2013; Gabbett, 2016; Pavin et al., 2019; Rey et al., 2019), we suggest a simple strategy of allowing many (e.g., unlimited) substitutions.

**Figure 1:**
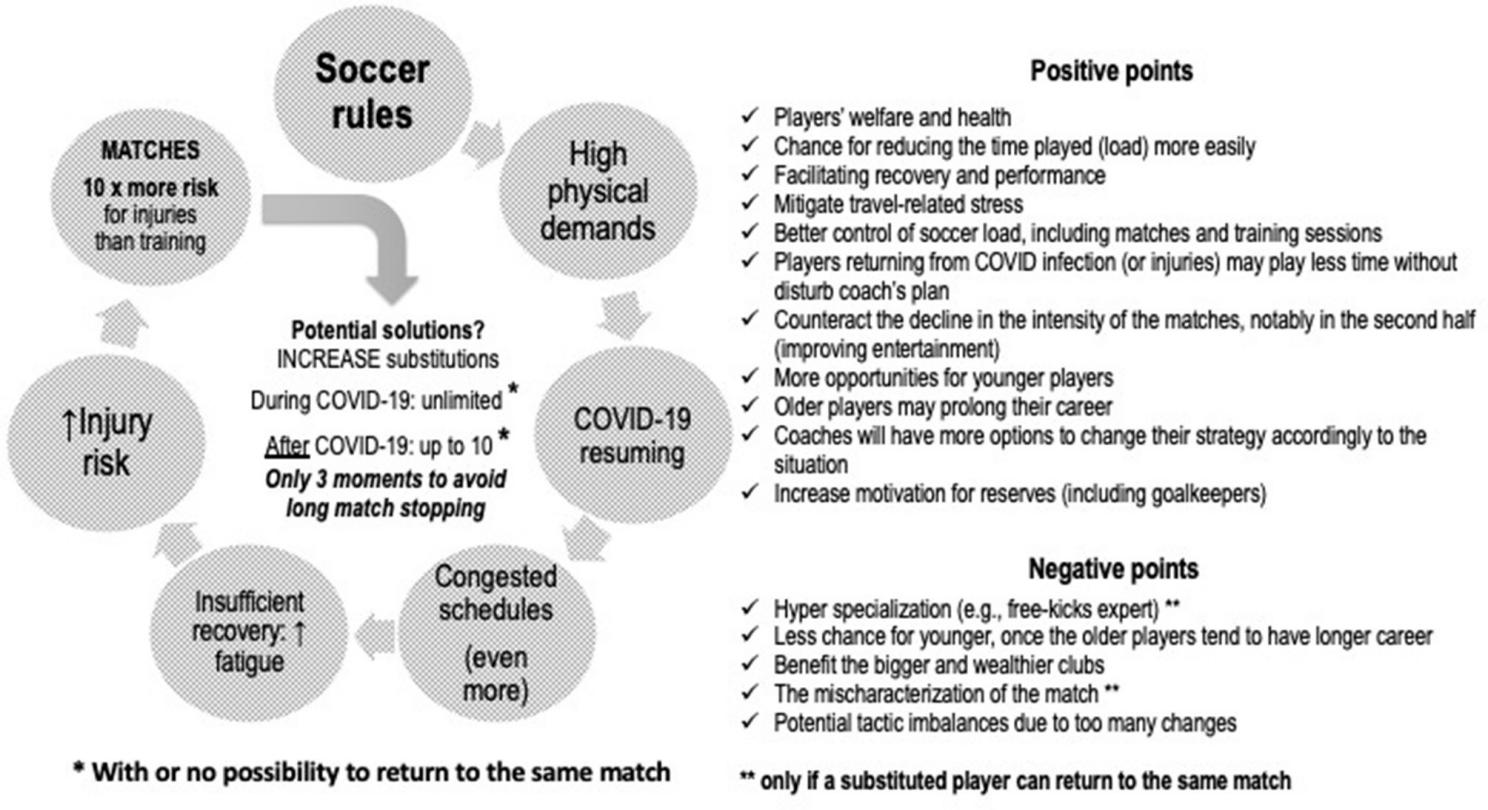
A theoretical scheme showing the relationship between soccer rules, physical demands, the matches resuming due to the COVID-19 pandemic break, and the injury risk. Potential positive and negative points if an increment in the number of substitutions allowed during the soccer match would happen permanently (regular rule) and particularly during the COVID-19 special calendars.

Fewer matches to increase time for proper recovery and better fitness preparation (Nédélec et al., 2013; Gabbett, 2016; Silva et al., 2018) or including more substitutions during the matches to make it possible to “dose” the load during matches. We believe fewer matches are essential for players' welfare. However, considering the real-world scenario, involving the COVID-19 overlap of matches, more substitutions might be necessary. Despite this suggestion, we acknowledge that changing rules may result in several other effects. Figure 1 shows the potential positive and negative points of allowing more substitutions.

## Rules, Physical Demands, and Substitutions

Normally, soccer coaches can make three substitutions. Due to the COVID-19 changes in the calendar season, up to five substitutions were allowed. During international and official matches, soccer coaches have 12 available substitute players. Therefore, the regular law of the game allows “only” ~13% of substitutions (3 players out of 23 available). This is too few compared, for example, with other popular invasive team sports (e.g., handball), which can replace 100% of players, with substituted players allowed to re-enter the game. The increase in soccer substitutions due to the COVID-19 (five players) increases the percentage to ~21.7%, but it is still small considering the high physical demands (Mota et al., 2020). Additionally, the substituted player cannot return to the match in soccer, even in the case of serious injury. An old study comparing injuries in soccer vs. handball reported that ~80% of soccer players continued to play when injured because the limit of substitutions was already met, presumably aggravating the injuries (Jørgensen, 1984). This situation tends to be even worse currently, as modern soccer is significantly more demanding (Barnes et al., 2014; Souglis et al., 2015; Silva et al., 2018). Recently the International Football Association Board (IFAB) has discussed concussion substitutes in soccer (FIFA.com, 2020c), which are in line with this article (e.g., safer and healthier sport).

Unlike other team sports (e.g., futsal), soccer has no time-outs, limiting the opportunity for brief recovery, and has the offside rule. To prevent offside, soccer players must move back and forth during the match. Such circumstances (i.e., restricted substitutions, no time-out, and offside law) overload soccer players even more during the matches. Considering that soccer matches have ~10 times higher injury rates than training sessions (López-Valenciano et al., 2020) and fatigue is associated with injuries (Ekstrand et al., 2004; Bengtsson et al., 2013; Gabbett, 2016; Silva et al., 2018), the increment (up to five substitutions) due to the COVID-19 seems insufficient.

A recent paper showed that substitutes who participated in the matches (non-starters) presented a lower workload, contemplating both matches and training sessions, during six matches in 21 days (i.e., congested schedules) (Gualtieri et al., 2020). These data suggest that matches are crucial in the training process, meaning that substitutes may be detrained. Typically, the coach has 12 substitutes players available, but only 25% of them (three) can participate in the match. The other nine (75%) could be investing the time to train and thus avoid detraining (Gualtieri et al., 2020) and prevent injuries (Gabbett, 2016). It is a poor use of time and demotivating for reserves players (Hills et al., 2018), and a waste of financial resources for the clubs (e.g., hotels, travels). A “game-changer” would be to increase soccer substitutions (Figure 1). This update on the rules is simpler and easier than other solutions, (e.g., fewer competitions) since economic impact would be another issue (Ahlert, 2001).

Increasing substitutions during a match, while keeping the current practice of allowing only three moments to avoid several stops in match action (Figure 1), might be a bright decision. If such changes do not happen now, especially during the COVID-19 resuming period, the issue of congested schedules (Bengtsson et al., 2013; Dellal et al., 2015), and consequently the incorporated fatigue and a higher risk of injuries (Bengtsson et al., 2013; Dellal et al., 2015), will continue. In addition to the possible health and financial benefits (i.e., high costs of an injured player) from increasing substitutions on soccer, it would probably improve match intensity, increasing the entertainment value for fans and sponsors, as substitute players covered a larger running distance at higher intensities (Carling et al., 2010; Bradley et al., 2014).

## Final Considerations

Although we are advocating an increase in substitutions, especially during the special COVID-19 calendar, we acknowledge that the regular calendar should be reviewed to avoid “kill the goose that lays golden eggs.” Also, according to Figure 1, only one point may have a deep impact (positive or negative). Therefore, such possibilities must be considered deeply implementing changes.

Considering that injury risk are much higher during matches (vs. training) (López-Valenciano et al., 2020) and that the soccer match itself is very demanding (Silva et al., 2018; Harper et al., 2019; Mota et al., 2020), increasing the number of substitutions in elite soccer is a simple strategy to mitigate relevant concerns such as high injury rates and congested schedules, especially through the COVID-19 pandemic. Implementing more substitutions in elite soccer may result in benefits in player welfare and great entertainment for fans and sponsors. A real game-changer!

## Author Contributions

GM and MM made substantial contributions to the conception, design, and drafting of the work, as well as the analysis and interpretation of data for the work. GM, IS, and MM revised it critically for important intellectual content, provided approval for publication of the content, and agreed to be accountable for all aspects of the work in ensuring that questions related to the accuracy or integrity of any part of the work were appropriately investigated and resolved. All authors contributed to the article and approved the submitted version.

## Conflict of Interest

The authors declare that the research was conducted in the absence of any commercial or financial relationships that could be construed as a potential conflict of interest.
